# Choroidal Morphologic and Vascular Features in Patients With Myopic Choroidal Neovascularization and Different Levels of Myopia Based on Image Binarization of Optical Coherence Tomography

**DOI:** 10.3389/fmed.2021.791012

**Published:** 2022-01-04

**Authors:** Xinglin Wang, Jiarui Yang, Yushi Liu, Luling Yang, Huaqin Xia, Xiaotong Ren, Qingyi Hou, Yimeng Ge, Changguan Wang, Xuemin Li

**Affiliations:** ^1^Department of Ophthalmology, Peking University Third Hospital, Beijing, China; ^2^Beijing Key Laboratory of Restoration of Damaged Ocular Nerve, Beijing, China

**Keywords:** choroidal structure, high myopia, myopic choroidal neovascularization (mCNV), choroidal vascularity index (CVI), optical coherence tomography

## Abstract

**Purpose:** To characterize the choroidal morphologic and vascular features in different levels of myopes and patients with myopic choroidal neovascularization (mCNV).

**Methods:** A total of 148 subjects were enrolled in this cross-sectional study, including 78 low-to-moderate myopes (LMM), 53 high myopes (HM), and 17 high myopic patients with mCNV. Ocular biometrics were measured using an optical low-coherence reflectometry device. Retinal and choroidal imaging was performed using enhanced depth imaging (EDI) spectral domain optical coherence tomography (OCT). Retinal parameters including retinal thickness and retinal volume were obtained from a built-in software. Binarization technique was adopted to investigate choroidal parameters including choroidal thickness (CT), vascular area, stromal area, and choroidal vascularity index (CVI). Choroidal parameters were measured at five locations to cover as much area of choroid as possible, and their patterns of distribution were further analyzed.

**Results:** Patients with mCNV had an atrophic retina of comparable thickness to HM (273.65 ± 17.28 vs. 276.49 ± 13.29 μm, *p* = 0.47), but the choroid was thinner than that of HM (153.94 ± 15.12 vs. 236.09 ± 38.51 μm, *p* < 0.001). Subfoveal CVI was greatest in the mCNV eyes (0.651 ± 0.009), followed by HM (0.645 ± 0.012) and LMM eyes (0.636 ± 0.012). Similar to CT, CVI was also found significantly different among these three groups at all five locations (*p* for trend < 0.001 for all locations). Axial length (AL) was negatively correlated with retinal volume (*r* = −0.236, *p* = 0.009), which is the only significant finding in associations between ocular factors and retinal parameters. Strong, negative correlations were identified between AL and subfoveal choroidal thickness (SFCT, *r* = −0.820, *p* < 0.001). However, AL was positively correlated with subfoveal CVI (*r* = 0.668, *p* < 0.001). CVI was greater in myopic eyes with thinner choroid (*r* = −0.578, *p* < 0.001). BCVA exhibited no significant association with CVI (*r* = 0.139, *p* = 0.092), but was negatively correlated with SFCT (*r* = −0.386, *p* < 0.001) and positively correlated with AL (*r* = 0.351, *p* < 0.001).

**Conclusion:** Choroid in patients with mCNV was thinner yet more vascularized than that in HM and LMM subjects. CVI increased with a longer AL which was associated with a smaller SFCT, choroidal vascular area (VA), and total choroidal area (TCA). Better BCVA was achieved in subjects with thicker SFCT and shorter AL.

## Introduction

Myopia is one of the most common ocular disease globally, with a prevalence of 10–30% in the adult population and 80–90% in young adults in East and Southeast Asia (1). Pathological myopia is one of the leading causes of visual impairment in the world, in which excessive axial elongation of the globe causes biomechanical stretching and thinning of choroid and retinal pigmented epithelium (RPE) layers (2–4), leading to increased risk of chorioretinal complications such as myopic choroidal neovascularization (mCNV), posterior staphyloma, lacquer cracks, and myopic foveoschisis, among which mCNV is considered to be sight-threatening with poor prognosis without treatment (5–10).

Recent development of enhanced depth imaging (EDI) optical coherence tomography (OCT) has enabled non-invasive, quantitative assessment of the choroid in myopia (11–13). A number of studies have found that with the progression of myopia, choroidal-related factors measured by OCT including choroidal thickness (CT) and choroidal vascular index (CVI) change significantly, indicating that the choroidal morphologic and vascular alterations may accompany the development of myopia (14–17). Reduction in choroidal circulation flow has been shown to occur in high myopia, which may be important in the pathogenesis of mCNV, and a reduction in CT in pathological myopic eyes with mCNV has been demonstrated using OCT imaging (18). Yet, to the best of our knowledge, these studies mostly focused on the choroidal capillary plexus density as shown in OCTA (19–21), or only identified total choroidal vascular density (including choroidal capillary plexus, Haller's and Sattler's layer of choroid) in subfoveal areas (16, 22). There is a lack of investigation on the variations of full-layer choroidal structure in different subretinal areas, and the role of choroidal vessel distributions in the pathogenesis of mCNV remains unclear.

The aim of our study was to compare the morphologic and vascular features of the choroid in patients with mCNV with those of different levels of myopia. Additionally, choroid was divided into five sectors to observe the CT and CVI variation in different regions and to characterize patterns of distribution of choroidal blood flow and their potential connections to the pathogenesis and clinical development of mCNV.

## Methods

### Study Population

This cross-sectional study was conducted in accordance with the tenets of the Declaration of Helsinki and was approved by the Ethical Committee of Peking University Third Hospital. Informed consent was obtained before enrollment. Myopic subjects without any previous significant ocular trauma or surgery and/or any clinically significant ocular comorbidity other than mCNV were enrolled and were further categorized into three groups based on their spherical equivalent (SE) and the presence of mCNV: (1) low-to-moderate myopes (LMM): low-to-moderate myopia [SE, −5.75 to −1.00 diopter (D)], and with-the-rule astigmatism no > −1.50D; (2) high myopes (HM): high myopia [SE, −6.00 diopter or worse (D)], and with-the-rule astigmatism no > −1.50D, and (3) mCNV group. The inclusion criteria of mCNV group were as follows: (1) newly developed active CNV confirmed with fundus fluorescein angiography (FFA), and (2) bilateral pathological myopia, defined as SE of < −6 diopters (D) in phakic patients (unless previously undergone refractive surgery) or axial length (AL) more than 26.5 mm, with typical degenerative changes in pathological myopia (19). Patients in mCNV group with Fuch's hemorrhage or CNV secondary to other causes other than pathological myopia, and subjects in the LMM/HM group with best corrected visual acuity (BCVA) higher than 0 [LogMAR], were excluded. We did not rule out subjects with the presence of peripapillary atrophy, lacquer crack, posterior staphyloma, or chorioretinal atrophy, as such changes are commonly seen in HM.

### Ophthalmic Examination and Measurements

All subjects underwent a standardized ophthalmologic examination including measurement of refractive error/SE, BCVA, intraocular pressure (IOP), and AL. Refractive error was screened with autorefraction (Canon Autorefractor RK-F1; Canon Inc. Ltd., Tochigiken, Japan) and confirmed with manifest refraction in which the BCVA was measured monocularly using a logarithm of the minimum angle of resolution (LogMAR) chart (Lighthouse International, New York, NY, USA) at a distance of 4 meters. Biometry measurements (i.e., AL, anterior chamber depth [ACD], and keratometry readings) were obtained from the non-contact Zeiss IOLMaster (V3.01; Carl Zeiss Meditec AG, Jena, Germany). Non-contact tonometry (Auto Non-Contact Tonometer, NT-3000; Nidek, Gamagori, Japan) was used for measuring IOP, and Goldmann applanation tonometry (Haag-Streit, Bern, Switzerland) was performed by study ophthalmologists if IOP was found to be 21 mmHg or more. Slit-lamp examination and dilated fundus examination were carried out in all subjects. Fundus photography was performed using retinal camera (Canon CR-DGi with a 10-DSLR back, Tokyo, Japan). FFA was performed in HM with susceptible subretinal hyperreflective material overlying the RPE on OCT to confirm the presence of mCNV (TRC-50X/IMAGEnet 2000; Topcon, Tokyo, Japan).

### OCT Imaging—Retinal and Choroidal Parameters

The retina and choroidal architectural parameters were determined using cirrus HD-OCT in EDI mode (Carl Zeiss Meditec Inc., Dublin, CA, USA). Choroid was imaged with EDI modality after pupil dilation. EDI is a method that improves resolution of choroidal detail by automatically setting the choroid closer to the zero-delay line and thus theoretically provides better visualization of the choroid scleral interface (CSI) than in standard retinal SD-OCT images (22). A 21-line 6-mm raster (0.3 mm between the lines, using the automatic averaging and eye-tracking features of the proprietary device) and a 512 × 128 macular cube scan (128 lines consisting of 512 A-scans and a central horizontal HD B-scan) centered onto the fovea of both eyes of each subject were obtained. Three horizontal lines passing through the center of the fovea and 1.5 mm superior and inferior to the fovea were selected for analysis.

Data of the retinal parameters including central thickness, volume cube, and average thickness were collected directly from the built-in 512 × 128 macular cube scan report. CT was measured as the distance between the Bruch membrane (located at the lower edge of the RPE) and the CSI, using the built-in calipers tool at five locations (subfoveal, 1.5 mm temporal, nasal, superior, and inferior to the fovea).

**Figure 1:**
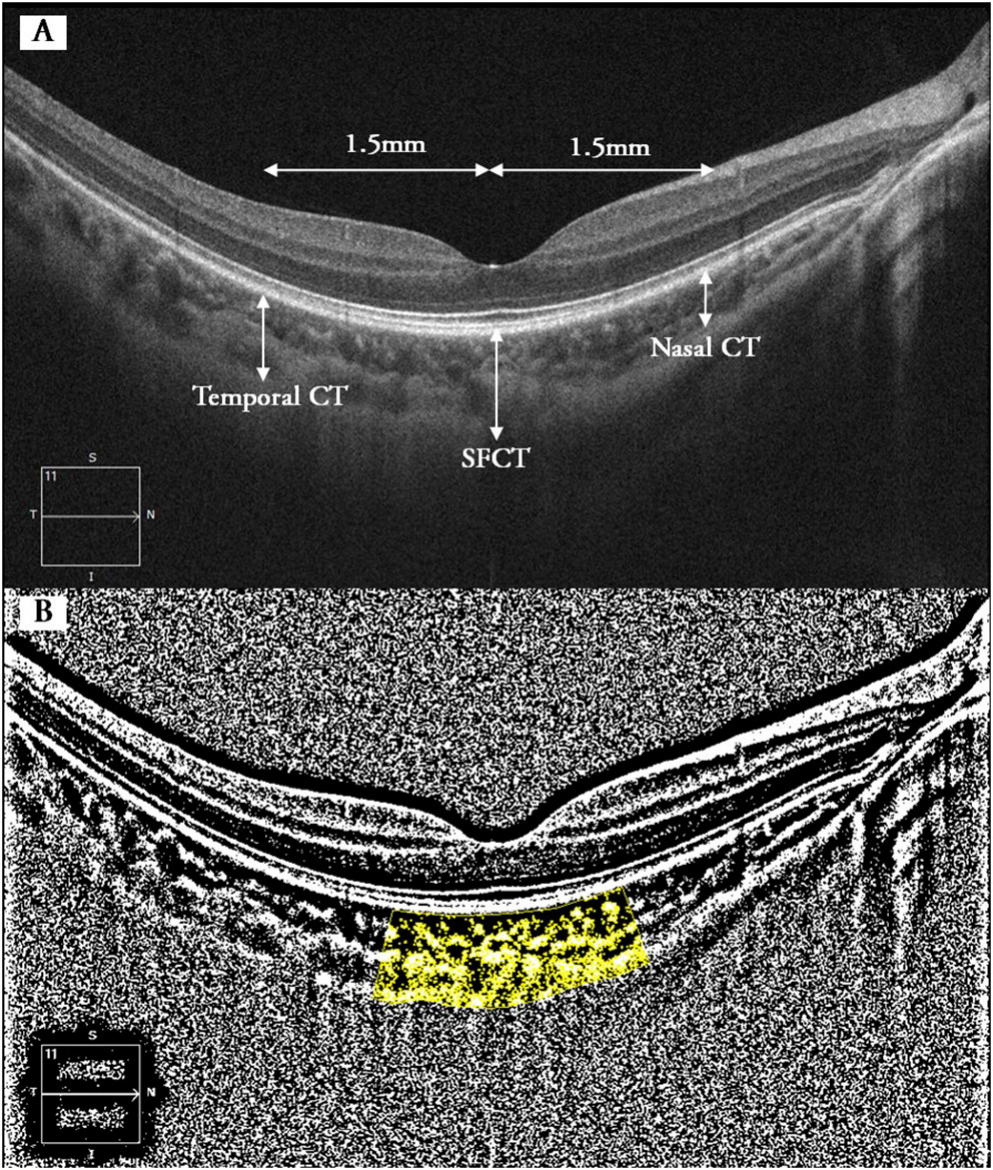
Binarization analysis of choroidal structure from OCT images. **(A)** Five locations where CT and CVI were measured (superior and inferior not shown). **(B)** Identification of the subfoveal choroidal segments using the binarization technique. Dark pixels refer to vascular components of choroid, whereas white pixels represent stromal components. CT, choroidal thickness; SFCT, subfoveal choroidal thickness; CVI, choroidal vascularity index.

Choroidal vascularity index was calculated manually by exporting all selected images with a 1:1 pixel ratio into ImageJ 1.7.0 software (National Institutes of Health, Bethesda, MD, USA) in which further measurement was taken. Briefly, the polygon tool was used to select the region of interest (choroid) in different areas with a length of 1.5 mm and centered at the same spots where retinal and CT were metered (Figure 1). After converting the image into eight bit, Niblack's auto-local threshold was applied to binarize the image and demarcate the choroidal vascular and stromal area (VA and SA, respectively). The total choroidal area (TCA), VA, and SA were measured and calculated as suggested previously (23). CVI was defined as the ratio of TCA divided by VA.

Binocular images were collected but only right eyes were chosen for subsequent analysis. In addition, we evaluated the intraobserver reliability of choriodal measurements at all five locations in three groups. All measurements were taken again by the same experienced clinician who was masked to subject characteristics and clinical diagnosis after an interval of 3 days. The average of the two measurements were recorded and used for further analysis.

### Statistical Analysis

Statistical analysis was performed using SPSS version 20.0 (SPSS, Inc, Chicago, IL), and *p* < 0.05 was considered to be statistically significant. All values were represented as mean ± SD or mean (SD), unless otherwise stated. The intrasession repeatability of the CT and CVI was measured by the absolute agreement model of the intraclass correlation coefficient (ICC) (24). The normality of the data was tested using the Kolmogorov–Smirnov test. One-way ANOVA test and chi-squared test were used to make comparisons among three or more groups for descriptive and categorical data, respectively, followed by Bonferroni post-test. Repeated-measures ANOVA with Bonferroni post-test was used to compare CT and CVI at various locations within each group. Independent samples *t*-tests were used for comparing the differences between two groups. Generalized linear model was used to assess the mean CT and CVI across different locations in eyes with varying degree of myopia, and conditions such as posterior staphyloma and chorioretinal atrophy were included to adjust for potential residual confounding. Pearson's correlation analysis was used to analyze the associations between OCT parameters and ocular factors.

## Results

### Patient Characteristics and Intraobserver Reliability

One hundred and sixty subjects were enrolled initially, including 80 LMMs, 60 HMs, and 20 mCNVs. We excluded 12 subjects because their choroidal images were not of optimal quality to perform accurate measurements of their choroidal traits, leaving 148 subjects with complete data for analysis. The demographics and ocular characteristics of the study population are shown in Table 1. Of all the subjects enrolled, only three participants in the mCNV group suffered from mild nuclear cataract of NO2NC2 according to LOCS III grading system. Among the HM and mCNV groups, 84.29% (59/70) had peripapillary atrophy, 27.14% (19/70) had posterior staphyloma, 12.86% (9/70) had chorioretinal atrophy, and 2.86% (2/70) had lacquer cracks. In terms of reliability of CT and CVI measurements, the intraobserver reliability for LMM, HM, and mCNV groups was excellent for all locations of choroidal parameters (Table 2).

**Table 1:** Baseline characteristics of study subjects.

	**LMM (*n* = 78)**	**HM (*n* = 53)**	**mCNV (*n* = 17)**	***p*** **-value**
Age, years	26.58 (0.56)	28.13 (1.63)	43.06 (1.91)	<0.001
Male/Female	41/37	29/24	10/7	0.89
Axial length, mm	24.95 (1.05)	26.42 (0.95)	28.08 (1.11)	<0.001
Spherical equivalent, D	−4.26 (1.35)	−7.62 (1.18)	−9.37 (1.79)	<0.001
BCVA, LogMAR	−0.27 (0.06)	−0.02(0.04)	0.10 (0.11)	<0.001
Intraocular pressure, mmHg	15.84 (3.02)	16.77 (5.33)	16.29 (3.02)	0.36

*Data presented are means (standard deviations)*.

*LMM, low-to-moderate myopia; HM, high myopia; mCNV, myopic choroidal neovascularization; BCVA, best-corrected visual acuity*.

**Table 2:** Intraobserver reliability of choroidal parameters in LMM, HM, and mCNV groups at different locations.

**Locations of measurement**	**LMM (*****n*** **=** **78)**		**HM (*****n*** **=** **53)**		**mCNV (*****n*** **=** **17)**	
	**ICC (95%CI)**	Mean difference^*^ (SD)	**ICC (95%CI)**	Mean difference^*^ (SD)	**ICC (95%CI)**	Mean difference^*^ (SD)
**CT (μm)**						
Subfoveal	0.98 (0.96–0.99)	9.42 (12.88)	0.96 (0.93–0.98)	8.27 (10.75)	0.95 (0.90–0.99)	10.32 (14.96)
Nasal, 1.5 mm	0.96 (0.93–0.98)	−9.85 (14.07)	0.96 (0.93–0.98)	9.11 (11.71)	0.95 (0.91–0.99)	−8.90 (13.77)
Temporal, 1.5 mm	0.97 (0.95–0.99)	8.31 (10.43)	0.97 (0.95–0.99)	10.32 (13.94)	0.96 (0.93–0.98)	10.41 (16.10)
Superior, 1.5 mm	0.96 (0.93–0.99)	9.28 (7.15)	0.96 (0.93–0.98)	−7.14 (8.41)	0.96 (0.93–0.98)	9.45 (6.15)
Inferior, 1.5 mm	0.96 (0.93–0.98)	10.17 (12.51)	0.96 (0.93–0.98)	8.58 (11.85)	0.98 (0.97–0.99)	10.27 (16.33)
**CVI**						
Subfoveal	0.96 (0.92–0.99)	0.018 (0.021)	0.97 (0.95–0.99)	0.014 (0.012)	0.96 (0.93–0.98)	0.019 (0.024)
Nasal, 1.5 mm	0.95 (0.90–0.99)	0.026 (0.015)	0.95 (0.90–0.99)	0.017 (0.025)	0.97 (0.96–0.99)	0.023 (0.016)
Temporal, 1.5 mm	0.95 (0.90–0.98)	−0.007 (0.013)	0.96 (0.93–0.98)	0.011 (0.010)	0.95 (0.91–0.99)	0.016 (0.017)
Superior, 1.5 mm	0.96 (0.93–0.98)	0.020 (0.041)	0.97 (0.96–0.99)	−0.009 (0.021)	0.95 (0.90–0.98)	0.018 (0.022)
Inferior, 1.5 mm	0.97 (0.95–0.99)	0.014 (0.012)	0.96 (0.92–0.99)	0.011 (0.012)	0.96 (0.93–0.98)	−0.015 (0.014)

*ICC, intraclass correlation coefficient; CI, confidence interval; SD, standard deviation. LMM, low-to-moderate myopia; HM, high myopia; mCNV, myopic choroidal neovascularization; CT, choroidal thickness; CVI, choroidal vascularity index*.

* *Mean difference was determined from the 1st measurement minus 2nd measurement*.

### Difference in OCT Parameters Among Three Groups

Table 3 presents retinal and choroidal morphological and vascular characteristics of eyes with different myopic conditions in each group. Significant difference was found in volume cube (*p* = 0.003) and retinal average thickness (*p* = 0.009) among these three groups whereas all groups had comparable retinal central thickness (*p* = 0.23, Table 3 and Figure 2A). However, HM eyes and mCNV eyes showed similar retinal characteristics in terms of retinal volume (9.96 ± 0.49 vs. 9.82 ± 0.43, *p* = 0.62), retinal average thickness (276.49 ± 13.29 vs. 273.65 ±17.28, *p* = 0.53), and retinal central thickness (246.29 ± 20.68 vs. 243.94 ± 21.27, *p* = 0.15). LMM eyes had thicker retinal average thickness and larger retinal volume than the other two groups of eyes (Table 3 and Figures 2B,C). Choroidal parameters, including TCA, choroidal vascular area (VA), choroidal stromal area (SA), and CT, decreased when subjects had a higher stage and worse condition of myopia (Table 3 and Figures 2D–G). Subfoveal CVI was greatest in the mCNV eyes (0.651 ± 0.009), followed by HM (0.645 ± 0.012) and LMM eyes (0.636 ± 0.012), as shown in Table 3 and Figure 2H.

**Table 3:** Retinal and choroidal morphological and vascular characteristics on OCT in LMM, HM, and mCNV eyes.

	**LMM (*n* = 78)**	**HM (*n* = 53)**	**mCNV (*n* = 17)**	***p*** **-value** ^**#**^
**Retinal parameters on OCT**				
Central thickness, μm	251.40 (17.16)	246.29 (20.68)	243.94 (21.27)	0.23
Volume cube, mm^3^	10.17 (0.37)	9.96 (0.49)	9.82 (0.43)	0.003
Average thickness, μm	282.63 (10.35)	276.49 (13.29)	273.65 (17.28)	0.009
**Choroidal parameters on OCT**				
Total choroidal area, mm^2^	2.58 (0.70)	1.82 (0.61)	1.00 (0.25)	<0.001
Vascular area, mm^2^	1.63 (0.42)	1.17 (0.38)	0.65 (0.16)	<0.001
Stromal area, mm^2^	0.95 (0.28)	0.65 (0.24)	0.35 (0.09)	<0.001
Choroidal thickness, μm	295.14 (41.52)	236.09 (38.51)	153.94 (15.12)	<0.001
CVI	0.636 (0.012)	0.645 (0.012)	0.651 (0.009)	<0.001

*Data presented are means (standard deviations)*.

*LMM, low to moderate myopia; HM, high myopia; mCNV, myopic choroidal neovascularization; OCT, optical coherence tomography; CVI, choroidal vascularity index*.

# *Based on one-way ANOVA*.

**Figure 2:**
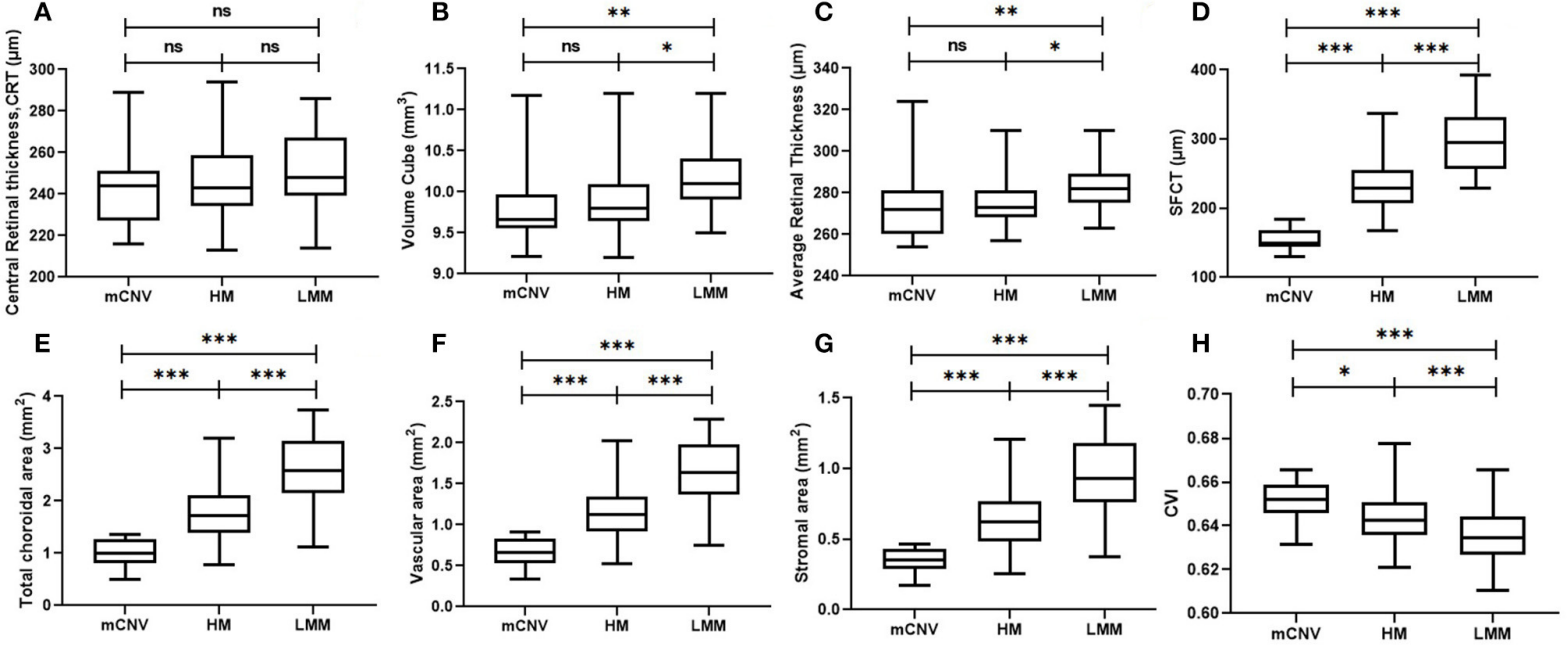
Retinal and choroidal morphological and vascular characteristics on OCT, including central retinal thickness **(A)**, retinal volume cube **(B)**, average retinal thickness (C), subfoveal choroidal thickness **(D)**, total choroidal area **(E)**, choroidal vascular area **(F)**, choroidal stromal area **(G)**, and CVI **(H)** in LMM (*n* = 78), HM (*n* = 53), and mCNV (*n* = 17) eyes. LMM, low-to-moderate myopia; HM, high myopia; mCNV, myopic choroidal neovascularization; OCT, optical coherence tomography; SFCT, subfoveal choroidal thickness; CVI, choroidal vascular index. ns, no significance; *p < 0.05; **p < 0.01; ***p < 0.001. *p*-value was calculated using independent *t*-test.

### Choroidal Structure Changes at Different Locations

Choroidal thickness varied significantly across these three groups at all the locations (*p* for trend < 0.001 for all locations, Table 4). Eyes with mCNV had the significantly thinnest choroid at all locations, followed by HM and LMM eyes. In mCNV and HM eyes, choroid was found to be thinnest at the nasal location, followed by the inferior, subfoveal, superior, and temporal locations (*p* < 0.001). In comparison, the choroid in LMM eyes was thinnest at the nasal location, followed by the inferior, temporal, subfoveal, and superior locations (*p* < 0.001). Similar to the CT, CVI was also found significantly different among these three groups at all five locations (*p* for trend <0.001 for all locations, Table 4). However, the variation in CVI at all locations in each group was insignificant.

**Table 4:** Distribution of CT and CVI at five different locations across the LMM, HM, and mCNV groups.

**Locations of measurement**	**LMM (*n* = 78)**	**HM (*n* = 53)**	**mCNV (*n* = 17)**	**Changes in choroidal parameters** **across 3 myopic groups**	
				**Beta**	**p for trend^†^**
**Choroidal thickness**, **μm**					
Subfoveal	295.14 (41.52)	234.55 (37.12)	153.94 (15.12)	−70.68	<0.001
Nasal, 1.5 mm	248.86 (39.27)	188.14 (44.20)	106.32 (16.17)	−71.27	<0.001
Temporal, 1.5 mm	294.26 (46.34)	251.07 (30.56)	169.54 (21.05)	−62.37	<0.001
Superior, 1.5 mm	303.21 (35.74)	236.09 (38.51)	161.43 (19.91)	−70.88	<0.001
Inferior, 1.5 mm	292.44 (31.45)	229.06 (42.20)	142.28 (17.97)	−75.07	<0.001
	*p* < 0.001^#^	*p* < 0.001^#^	*p* < 0.001^#^		
**CVI**					
Subfoveal	0.6355 (0.1208)	0.6446 (0.0120)	0.6513 (0.0090)	0.0079	<0.001
Nasal, 1.5 mm	0.6353 (0.0306)	0.6440 (0.0148)	0.6500 (0.0109)	0.0074	<0.001
Temporal, 1.5 mm	0.6356 (0.0148)	0.6415 (0.0281)	0.6425 (0.0148)	0.0035	0.0072
Superior, 1.5 mm	0.6354 (0.0279)	0.6441 (0.0193)	0.6512 (0.0184)	0.0079	<0.001
Inferior, 1.5 mm	0.6355 (0.0923)	0.6443 (0.0378)	0.6488 (0.0469)	0.0067	<0.001
	*p* = 0.806^#^	*p* = 0.344^#^	*p* = 0.409^#^		

*Data presented are mean (standard deviations), adjusted for the presence of posterior staphyloma and chorioretinal atrophy*.

† *Generalized linear model, adjusted for the presence of posterior staphyloma and chorioretinal atrophy*.

# *Repeated-measures ANOVA, comparing the distribution of CT across subfoveal, nasal (1.5 mm), temporal (1.5 mm), superior (1.5 mm), and inferior (1.5 mm) locations*.

### Correlations Between Ocular Factors and OCT Parameters in All Myopes

**Figure 3:**
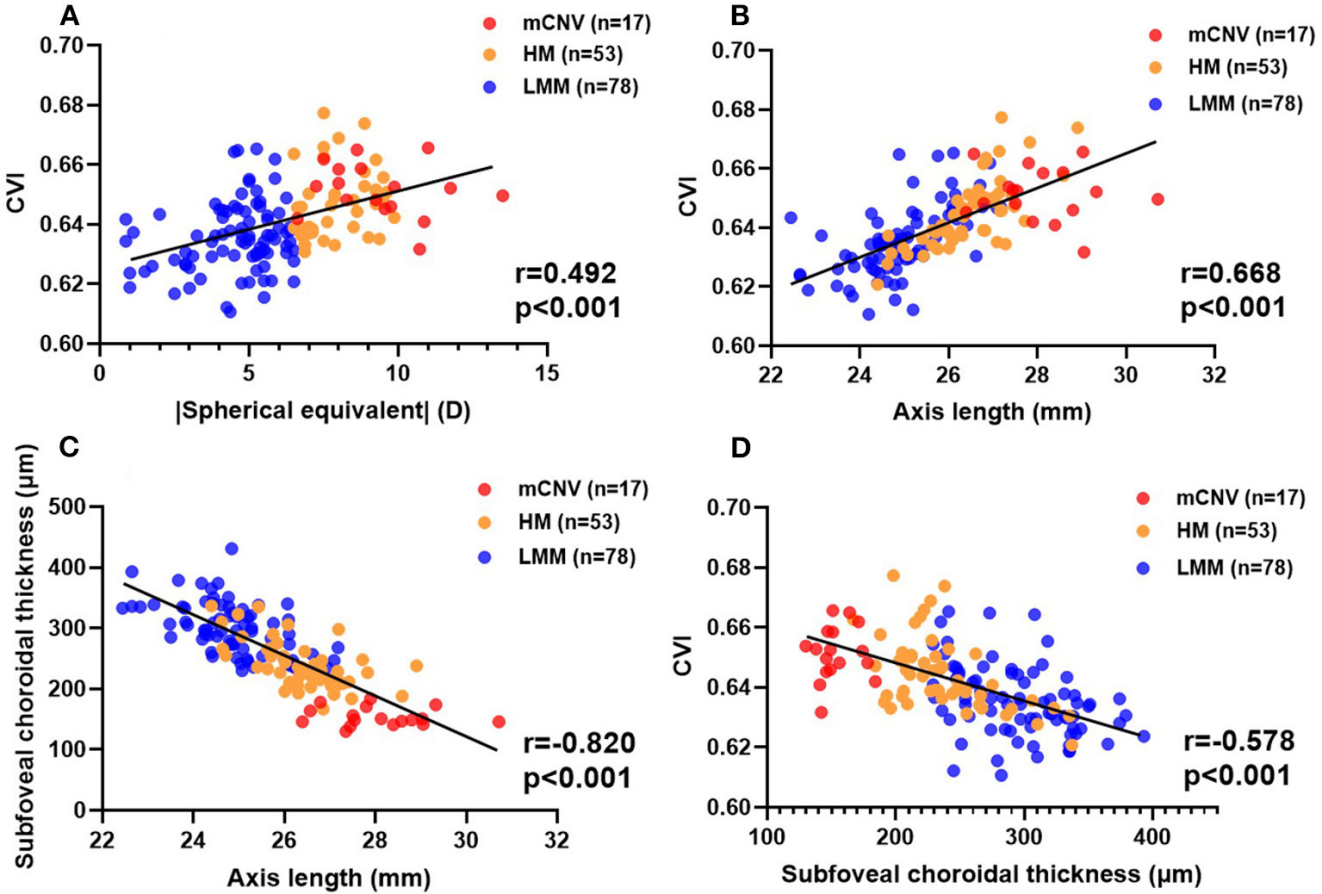
Relationship between some ocular factors and OCT parameters of the choroid. Scatter plots showing the associations between CVI and |SE| **(A)**, CVI and AL **(B)**, SFCT and AL **(C)**, and CVI and SFCT **(D)**. LMM, low-to-moderate myopia; HM, high myopia; mCNV, myopic choroidal neovascularization; SFCT, subfoveal choroidal thickness; CVI, choroidal vascular index.

Ocular factors including AL, SE (as all subjects were myopic, the absolute value of SE was applied), IOP, and BCVA were analyzed for their correlations with OCT parameters mentioned earlier, including retinal central thickness, retinal volume, retinal average thickness, SFCT, TCA, choroidal VA, choroidal SA, and CVI at subfoveal location (Figures 3A–D). IOP was not correlated with any of these OCT parameters (data not shown). AL was negatively correlated with retinal volume (*r* = −0.236, *p* = 0.009), which is the only significant finding in associations between ocular factors and retinal parameters. Strong, negative correlations were identified between AL and SFCT (Figure 3C, *r* = −0.820, *p* < 0.001), TCA (*r* = −0.857, *p* < 0.001), VA (*r* = −0.860, *p* < 0.001), and SA (*r* = −0.849, *p* < 0.001). However, AL was positively correlated with subfoveal CVI (Figure 3B, *r* = 0.668, *p* < 0.001). CVI was greater in myopic eyes with thinner choroid (Figure 3D, *r* = −0.578, *p* < 0.001). These results indicate that the increasing intraocular compression against fundus in eyes with long AL contributes to a thinner yet more vascularized choroid with less vascular and stromal choroidal area. BCVA was not correlated with CVI (*r* = 0.139, *p* = 0.092), but was negatively correlated with SFCT (*r* = −0.386, *p* < 0.001) and positively correlated with AL (*r* = 0.351, *p* < 0.001).

## Discussion

Myopia is not only a very common abnormal refractive condition, but also a potential sight-threatening ophthalmic disease when staged into its severe form (pathologic myopia). To our knowledge, our study here is the first comprehensive research on morphology and vasculature of the retina and choroid in different levels of myopes and pathological myopic eyes with mCNV. We discovered that both HM and mCNV eyes presented a thinner retina with a thinner yet more vascularized choroid than LMM. Although the morphology of retina was comparable between HM and mCNV eyes, mCNV eyes showed significant increase in CVI and reduction in both choroidal vascular and stromal area, which indicates the atrophy of choroidal components may lead to the pathologic state of myopia.

We adopted the binarization technique to manually measure the CT and differentiate the luminal (vascular) and stromal components of the choroid in OCT images. On the other hand, all retinal-related parameters were automatically acquired by the commercial OCT built-in software. To balance the comprehensiveness of our assessment of the choroid and labor workload, we selected and covered five different spots or areas on OCT images. The great intraobserver reliability at different locations among all subjects laid a solid foundation for our subsequent analysis.

In this study, mCNV and HM eyes were both high myopic (*SE* ≤ −6.0D), but mCNV group had a higher AL than HM group (28.08 ± 1.11 vs. 26.42 ± 0.95 mm, *p* < 0.001), suggesting that the continuous growth of AL may lead to pathologic state of high myopia (1). Subjects with high myopia or mCNV possessed a thinner retina with less volume and average thickness than that of LMM subjects. This is in accordance with previous studies regarding the alterations of retinal thickness and volume in different levels of myopia (11, 25, 26). However, all retinal parameters on OCT did not differ between HM and mCNV eyes. This implies that atrophy of retina, which is a pathological condition in HM, may have limited association with the onset of mCNV and is not a recommended parameter to distinguish mCNV and HM eyes. Previous studies have yielded that myopic eyes had thinner choroid than emmetropic eyes (16), but choroidal morphology and vascular characteristics were left undiscovered in mCNV eyes. In our study, we found that compared with LMM and HM eyes, CT, TCA, choroidal VA, and choroidal SA at all locations decreased the most in mCNV eyes. The consistent change in choroidal parameters as degree of myopia deepens suggests that the progressive choroidal thinning should be of greater concern to early detect and predict the onset of mCNV in HM. Patients with mCNV had the highest CVI among three groups whereas both the numerator (choroidal VA) and denominator (TCA) of CVI decreased in mCNV eyes. These findings reveal that mCNV eyes suffered from a higher level of choroidal atrophy than HM eyes, with relatively greater reduction in the stromal component compared with vascular components, which was further supported by the strong correlation between CVI and AL. However, effects of choroidal atrophy on the pathogenesis of pathologic myopia and mCNV remain to be elucidated.

Anatomic studies demonstrate that choroid plays a crucial role in supporting photoreceptor function; such atrophy may have significant deleterious impact on outer retina and RPE health. Previous studies have put forth the theory that alterations in the choroidal blood perfusion may lead to degenerative changes in pathological myopia and high myopia (2). Narrowing and loss of large choroidal vessels, and occlusion of choriocapillaris, have been suggested to be important in the pathogenesis of chorioretinal atrophy in pathological myopia (25). Dramatic reductions in CT and choroidal circulation have been observed in highly myopic eyes (27). Ischemia and vascular changes have also been proposed to be important factors in the pathogenesis of lacquer cracks and mCNV (26). Our discoveries may add important implications in the pathogenesis and possibly treatment response and may help to better understand choroidal involvement in high myopic and mCNV eyes.

Choroid was thickest superiorly and thinnest nasally in LMM eyes, which agrees with the previous studies (22, 28) and can be explained by two possible reasons that the choroidal watershed and the fetal choroidal fissure closes inferiorly at 7 weeks (29). In HMM and mCNV eyes, the thickest and thinnest choroid was at temporal and nasal locations. It is likely that the presence of posterior staphyloma, of which the most common types involve the macula and optic nerve regions (10, 30), may contribute to the thinning of the nasal choroid and the temporal shift in thickest point in high myopic eyes (31). The variation in CVI at all locations in each group was insignificant, indicating that the atrophy of vascular and stromal components covered entire choroid with comparable extent.

In addition to our finding that thinning choroid is a structural feature of high myopia, our results reveal that the extent of choroidal thinning and vascularization is significantly correlated with the magnitude of refractive error and prolongation of AL. Interestingly, CVI was greater in myopic eyes with thinner choroid which can be probably interpreted as a choroidal structural compensation mechanism to provide more oxygen and nourishment against the reduction in total blood flow caused by atrophy of choroid.

In contrast to Gupta's study (22) that demonstrated no correlation between choroidal parameters and visual acuity, we found a significant association between SFCT and BCVA. The discrepancy may be due to differences in study participants since we enrolled all levels of myopes (especially patients with mCNV with thinner choroid whose vision has already been compromised), whereas Gupta's study only covered HM and emmetropes. Interestingly, no such correlation was found between CVI and BCVA. Based on these findings, we speculated that a thin choroid, even though more vascularized, delivers decreased amounts of oxygen and nutrients to the retina, thus potentially affecting signal generation from the photoreceptors or cause loss of the overlying photoreceptors as a consequence.

There are several limitations of our study that needs to be addressed. First, our study has a relatively small sample size with <20 patients with mCNV and 40 HM recruited. Second, the cause–effect relationship cannot be ascertained owing to the cross-sectional nature of our study. Further endeavors validating the alteration of choroidal morphology and blood perfusion during progress of pathological myopia are needed. Third, The difference in AL and age between mCNV and HM groups cannot be ignored since older age and longer AL have been proven to be associated with thinner choroid (31). Here in our study, age was not associated with variation in CVI (*r* = 0.089, *p* = 0.237) and showed no correlation with SFCT in the multiple linear regression analysis (β = −0.023, 95% CI: −1.039–0.667, *p* = 0.667). However, to evaluate and compare the choroidal characteristics in HM and pathologic myopes more accurately and convincingly, upcoming studies should attempt to enroll AL- and age-matched subjects with high myopia and mCNV, respectively.

In conclusion, this study characterized choroidal structural and vascular features and patterns of distribution of choroidal blood flow in patients with mCNV, whose choroid was thinner yet more vascularized than that in HM and LMM subjects. CVI increased with a longer AL, which was associated with smaller SFCT, VA, and TCA. Better BCVA was achieved in subjects with thicker SFCT and shorter AL. The technique used to evaluate the choroids of mCNV should be applied in future investigations of mechanisms and interventions of pathological myopia

## Data Availability Statement

The raw data supporting the conclusions of this article will be made available by the authors, without undue reservation.

## Ethics Statement

The studies involving human participants were reviewed and approved by Institutional Review Board of Peking University Third Hospital. The patients/participants provided their written informed consent to participate in this study.

## Author Contributions

XL and CW: supervised the project. XW and JY: developed the original idea and wrote the manuscript. XW, YL, and LY: conducted the statistical analysis. XW, HX, XR, QH, and YG: collected the data. All authors contributed to the article and approved the submitted version.

## Funding

This study was supported by Natural Science Foundation of Beijing, China (grant number 7202229).

## Conflict of Interest

The authors declare that the research was conducted in the absence of any commercial or financial relationships that could be construed as a potential conflict of interest.

## Publisher's Note

All claims expressed in this article are solely those of the authors and do not necessarily represent those of their affiliated organizations, or those of the publisher, the editors and the reviewers. Any product that may be evaluated in this article, or claim that may be made by its manufacturer, is not guaranteed or endorsed by the publisher.
